# Thermo-Responsive Polymer Brushes with Side Graft Chains: Relationship Between Molecular Architecture and Underwater Adherence

**DOI:** 10.3390/ijms20246295

**Published:** 2019-12-13

**Authors:** Ugo Sidoli, Hisaschi T. Tee, Ivan Raguzin, Jakob Mühldorfer, Frederik R. Wurm, Alla Synytska

**Affiliations:** 1 Leibniz-Institut für Polymerforschung Dresden e.V., Hohe Straße 6, 01069 Dresden, Germany; sidoli@ipfdd.de (U.S.); ivanraguzin@gmail.com (I.R.); mail@jmuehldorfer.de (J.M.); 2 Technische Universität Dresden, 01062 Dresden, Germany; 3 Max Planck Institute of Polymer Research, Ackermannweg 10, 55128 Mainz, Germany; Hisaschi.Tee@outlook.de (H.T.T.); wurm@mpip-mainz.mpg.de (F.R.W.)

**Keywords:** underwater adhesion, atomic force microscopy, polymer brushes, responsive thin films

## Abstract

During the last few decades, wet adhesives have been developed for applications in various fields. Nonetheless, key questions such as the most suitable polymer architecture as well as the most suitable chemical composition remain open. In this article, we investigate the underwater adhesion properties of novel responsive polymer brushes with side graft chain architecture prepared using “*grafting through*” approach on flat surfaces. The incorporation in the backbone of thermo-responsive poly(*N*-isopropylacrylamide) (PNIPAm) allowed us to obtain LCST behavior in the final layers. PNIPAm is co-polymerized with poly(methyl ethylene phosphate) (PMEP), a poloyphosphoester. The final materials are characterized studying the surface-grafted polymer as well as the polymer from the bulk solution, and pure PNIPAm brush is used as reference. PNIPAm-g-PMEP copolymers retain the responsive behavior of PNIPAm: when T > LCST, a clear switching of properties is observed. More specifically, all layers above the critical temperature show collapse of the chains, increased hydrophobicity and variation of the surface charge even if no ionizable groups are present. Secondly, effect of adhesion parameters such as debonding rate and contact time is studied. Thirdly, the reversibility of the adhesive properties is confirmed by performing adhesion cycles. Finally, the adhesive properties of the layers are studied below and above the LCST against hydrophilic and hydrophobic substrates.

## 1. Introduction

Adhesive materials have a huge impact on our everyday routine. Simple actions, such as holding objects in our hands, could not take place without adhesion phenomena. Moreover, life itself relies on adhesion at different length scales, for instance, adhesion between cells or tissues. In the last few decades, wet adhesives found more and more applications due to their versatile nature in several industrial fields, including automotive and aerospace industries, as well as electronics and medicine.

Inspiration for these new materials comes from many examples of natural organisms showing remarkable adhesion in presence of water against native surfaces with little, if any, surface preparation [1,2]. The most studied and well-known candidate for underwater adhesion is the marine mussel. They are able to strongly bond to fouled surfaces using their byssal foot, where they produce adhesive proteins [1]. The exact mechanism involved in mussel adhesion is still unclear, but it has been demonstrated that these proteins contain a variety of chemical functionalities, being particularly rich in 3,4-dihydroxyphenylalanine (l-DOPA), amino groups, and phosphates [2]. This variety of functionalities allows the mussel to provide different kind of interactions, therefore promoting adhesion against several organic and inorganic surfaces [3]. Another sessile organism known for its underwater adhesion is the sandcastle worm. This animal is able to build its shell made of sand grains and mineral particles using a rapid-set glue or cement; in-depth studies pointed out that the components of this glue are highly charged proteins, mainly anionic polyphosphates and cationic polyamines [4,5]. When these charged proteins are mixed together, a phase separation occurs due to complex coacervation between oppositely charged polyelectrolytes. This phase separation, which is triggered by environmental conditions such as the ionic strength of the surrounding environment, allows the material to undergo a transition from fluid-like to a foamy porous solid. The successive curing phase, triggered by enzymes, provides hardening of the material and therefore enhanced cohesion [6]. The widespread studies of natural systems have given new inspiration and strategies in the design of bio-mimetic and bio-inspired wet adhesive materials. Due to the knowledge derived from the mussels and the sandcastle worm, the current research in this field can be divided in two main approaches: DOPA-based adhesives and coacervate-based adhesives. After the first publication of Messersmith and coworkers about the design of polydopamine coatings [7], a number of research groups tried to combine DOPA fragments with different functionalities in order to enhance the adhesive performances in wet conditions [8,9]. Later, DOPA has been combined with thermo-responsive *N*-isopropylacrylamide adding temperature as a trigger to make adhesive behavior responsive [10]. In the second approach, the concept of complex coacervation has been used to develop synthetic adhesives for bio-medical applications [11]. Stewart and coworkers used bio-inspired polyphopshates and polyamines to produce injectable coacervates which undergo a phase transition after injection into physiological environment [12]. These two approaches can also be combined, using DOPA as component for the coacervate as shown by Waite and coworkers [13].

Nonetheless, several adhesives show lower performances or reduced stability in a moist environment due to several phenomena such as erosion, swelling or change of the bulk mechanical properties due to moisture uptake [14]. Furthermore, non-specific and uncontrolled adhesion in wet environment is not always desirable, especially in biomedical applications [15]. For these reasons, there is a demand for materials which are able not only to provide strong and reliable adhesion underwater and in wet conditions, but also to control and tune the adhesion against different substrates. Using reversible bonds, materials able to switch between a highly adhesive state and a non-adhesive state can be obtained. Several systems have been proposed as a model for fundamental understanding of the behavior of adhesive and responsive polymer layers; polymer brushes are one of them [16,17]. Specifically, the possibility to combine different chemistries, compositions and architectures makes polymer brushes suitable for decreasing, tuning, or enhancing the adhesion against plenty of inorganic and organic substrates [18]. In the case of anti-fouling coatings, neutral hydrophilic [19] and electrically charged zwitterionic brushes [20,21] were shown to be effective against non-specific adhesion of cells and proteins as they are able to form a hydration layer at the surface that helps preventing the adsorption of proteins. As promoters of adhesion, brushes have been tested both in dry [22] and underwater [23,24] conditions, showing that the composition and wettability of the brushes are key parameters to obtain adhesion against hydrophilic or hydrophobic substrates. Moreover, the conditions of the measurement (pH, ionic strength, etc.) affect the performance of the polymer layer [24]. Furthermore, biomedical applications with the necessity of switchable adhesion can be considered by combining thermo-responsive and biocompatible building blocks [25]. Polyphosphoesters (PPEs) are a promising material class for biomedical applications due to their biodegradability and biocompatibility, diversified structures and versatile functionalities making them good alternative to other synthetic hydrophilic polymers like PEG, PHPMA, and others [26]. Similar to PEG, a common hydrophilic material for polymer brushes, hydrophilic PPEs like poly(ethyl ethylene phosphate) (PEEP) or poly(methyl ethylene phosphate) (PMEP) showed to be effective against non-specific adhesion of proteins [27,28]. Recently, our group studied the effect of composition and architecture of the polymer chain on the overall adhesive behavior [29,30]. Results pointed out how the performances of the adhesive layer are indeed affected by the ratio between hydrophilic and hydrophobic monomers, and also that the properties can be tuned by keeping the same chemical composition with different architectures (block, random copolymers). Despite the growing number of studies on gels, brushes, and other model systems, many fundamental questions remain open. For instance, the relationship between elasticity, interfacial charge density and the magnitude of the adhesion underwater still remains unclear. Similarly, how the degree of functionalization of a surface affects the final underwater adhesive properties is still to be addressed. Finally, it is still unknown what are the main mechanisms by which intermolecular forces in wet environment result in macroscopically measurable adhesion.

**Figure 1:**
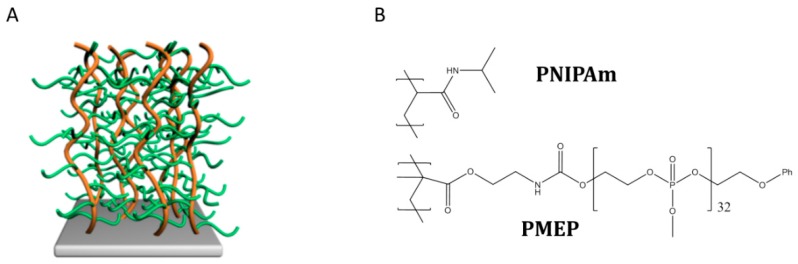
PNIPAm-g-PMEP polymer brush with side graft chain architecture (**A**) with PNIPAm backbone in orange and PMEP side-chains in green; and (**B**) composition.

In this article, we aim to fundamentally understand how the degree of functionalization in thermo-responsive grafted polymer brushes affects the underwater adhesive properties as well as the swelling behavior, polarity, and surface charge. For this, we have designed copolymer brushes with side graft chain architecture (Figure 1A) and varied chemical composition, resulting in different amounts of side-chains along the polymer backbone. The layers are prepared on flat substrates using a ‘grafting through’ approach, and the final copolymers carry respectively 5% and 10% of polar polyphosphates compared to the PNIPAm functionalities. The incorporation in the backbone of thermo-responsive units allowed us to obtain LCST behavior in the final layers. Firstly, the thermo-responsive behavior of the brushes in aqueous solutions is characterized, following the variation in thickness, wettability, and surface charge. Secondly, AFM-CP is used to evaluate the effect of key parameters for adhesion such as debonding rate and contact time against a SiO_2_ colloidal probe; moreover, the reversibility of the adhesive properties has been studied by performing cycles above and below the LCST. Thirdly, the adhesion properties of the layers are tested against hydrophilic (SiO_2_ and 3-Aminopropyltriethoxysilane) and hydrophobic (1H,1H,2H,2H- Perfluoro- octyldimethylchlorosilane) substrates with different wettability and surface charge.

## 2. Results and Discussion

### 2.1. Synthesis of PPE Macromonomer

**Figure 2:**
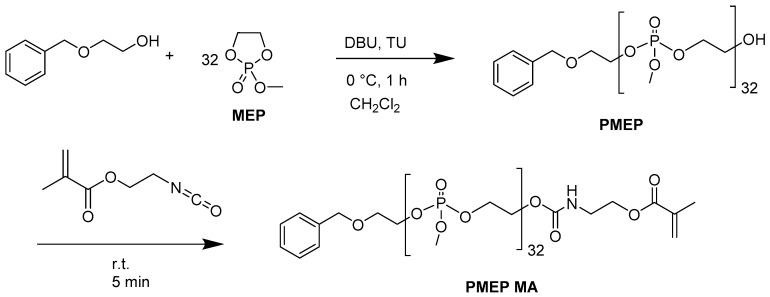
Polymerization of MEP and termination towards PMEP MA.

Poly(methyl ethylene phosphate) (PMEP) was synthesized by ring-opening polymerization (ROP, Figure 2) of the five-membered 2-methoxy-1,3,2-dioxaphospholane 2-oxide heterocycle with the presence of 1,8-diazabicyclo(5.4.0) undec-7-ene (DBU)/1-(3,5-bis (trifluoromethyl)phenyl)- 3-cyclohexylthiourea (TU) as catalyst/cocatalyst system and 2-(benzyloxy)ethanol as initiator. This polymer is known for a while [31], however compared to poly(ethyl ethylene phosphate) (PEEP) it was not used for further studies or applications. PEEP has been used in various studies as water-soluble segments in hydrogels, nanoassemblies, and nanocarriers, mainly as a degradable analogue for PEG [27,32,33]. However, by using PMEP, segments can be introduced which surpass the hydrophilicity of PEEP and even PEG, which has a distinguish effect on many properties like the swelling behavior. For the design of the polymerization macromere, the methacrylate end-groups were introduced by termination of ROP with 2-isocyanatoethyl methacrylate. The resulting functional and hydrophilic poly(methyl ethylene phosphate) methacrylate (PMEP MA) precursor was then able to be grafted to the substrate.

### 2.2. Synthesis of Graft PNIPAm-g-PMEP Polymer Brushes

**Figure 3:**
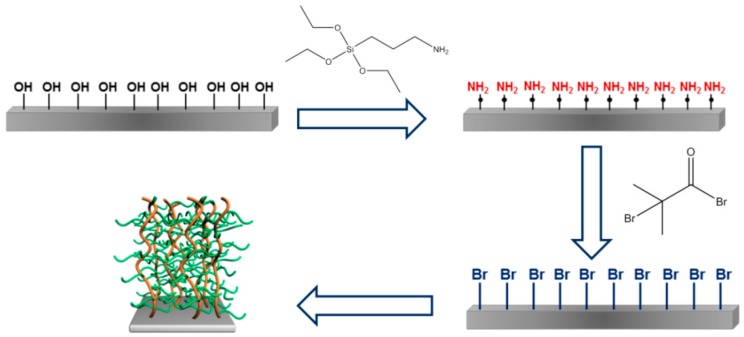
Wafer modification (APTES in red and BrIn in blue) and synthesis of PNIPAm-g-PMEP brush with side graft chain architecture (PNIPAm backbone in orange and PMEP side-chains in green)**.**

The graft PNIPAm-g-PMEP polymer brushes were synthetized via combination of the so called ‘grafting from’ and ‘grafting through’ approaches (Figure 3). The reference sample (PNIPAm) was produced by ‘grafting from’: here, a low molecular weight monomer is radically polymerized from the surface due to the surface-bound initiator, resulting in the formation of polymer brushes [34]. The copolymers were produced by ‘grafting through’: a low molecular weight monomer is radically copolymerized with a methacrylate macromonomer, permitting the incorporation of both monomers into the backbone [35].

Briefly, the silicon oxide layer was modified with 3-aminopropyl-triethoxysilane (APTES) producing amino groups, which were then used for the immobilization of α-bromoisobutyryl bromide (BrIn), the initiator for further polymerization [36]. The brushes were then produced by ARGET-ATRP using CuBr_2_ as catalyst, PMDTA as ligand for copper ions and ascorbic acid as reducing agent [17]. Each step of surface modification was proven by null-ellipsometry (Table 1).

**Figure 4:**
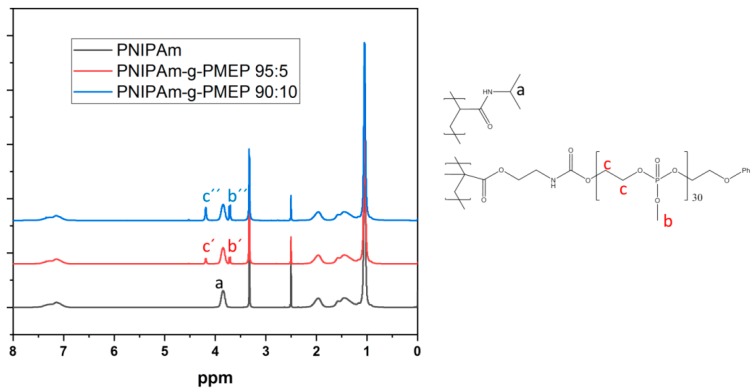
^1^H NMR of the synthetized brushes (PNIPAm in black, PNIPAm-g-PMEP 95:5 in red, PNIPAm-g-PMEP 90:10 in blue); the signals from PNIPAm a and from the oxomethyl groups b were used to calculate the polymer composition; solvent: CDCl_3_.

Each wafer was measured in three different spots in order to prove the homogeneity of every new layer. The overall thickness of the modified layers before polymerization was found to be less than 3 nm. A small amount of ethyl α-bromoisobutyrate initiator was added into the reaction mixture before polymerization. Analysis of the resulted polymer from the bulk solution was used to estimate the molecular weight and the composition of the polymer at the surface. Here, a reasonable assumption was made: the rates of polymerization at the substrate surface and in the bulk have comparable orders. Hereby, two grafted copolymers with different amounts of PMEP phosphate were produced, along with a reference sample made of pure PNIPAm. The composition of the copolymers was determined by NMR on the bulk polymer, as shown in Figure 4. Here, the peaks arising from the incorporation of the macromonomer can be seen in the red and blue spectra. The values 95:5 and 90:10 indicate the molar ratio between PNIPAm monomer units and methyl-phosphate functionalities (R-PO_3_-OMe-R). NMR spectra with integration values can be found in the Supplementary Materials.

The determination of the molecular weight and the dry thickness was then used for the calculation of the grafting density of the brush layer (Table 2); for a complete discussion concerning the grafting density, see the Supplementary Materials.

The average grafting density is around 0.2 chains/nm^2^, which is in good agreement with the values of polymer brushes synthetized using the same approach [25,29]. Since the distance between grafted chains is one order of magnitude smaller compared to the thickness of the polymer film (2 nm compared to 30 nm), our layer can in first approximation be considered in the brush regime [25]. Interestingly, both copolymers show smaller molecular weight, but higher thickness compared to the homopolymer. We suppose that the presence of sterically hindered side-chains forces the brushes to stretch, therefore resulting in higher thickness compared to the homopolymer (35 and 32 nm respectively compared to 30 nm). Nonetheless, we do not have an explanation yet for the fact that the copolymer 95:5, having smaller molecular weight than the copolymer 90:10, shows slightly higher thickness compared to the copolymer 90:10.

### 2.3. Determination of Turbidity/Cloud Point

**Figure 5:**
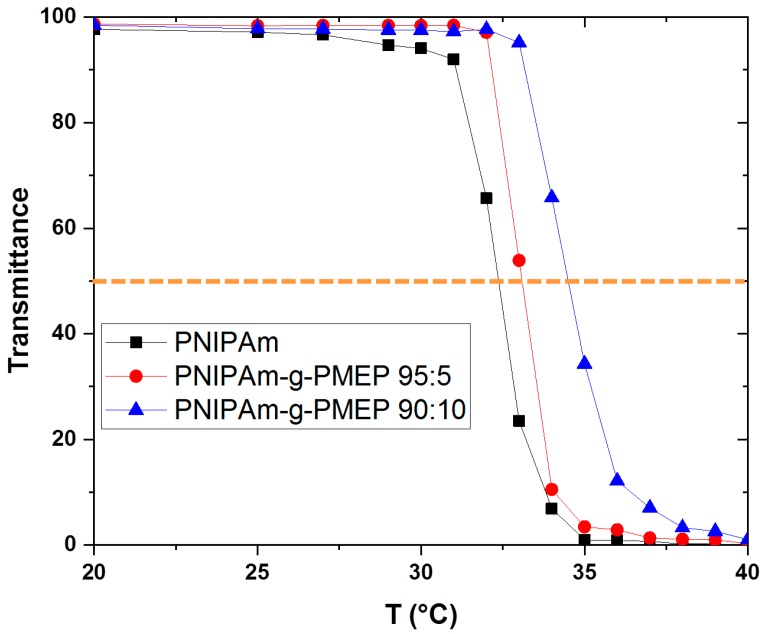
Cloud point as function of temperature in DI water (black: PNIPAm; red: PNIPAm-g-PMEP 95:5; blue: PNIPAm-g-PMEP 90:10).

After confirming the brush-like arrangement, we investigated the thermo-responsive behavior of the grafted polymers. In order to analyze the LCST temperature of the synthetized brushes, we studied the cloud point of the polymers from the bulk solution. The cloud point is an experimental parameter used to gain information about the LCST of a polymer, and it can be defined as the temperature at which the solution becomes turbid. Using UV–vis spectroscopy, the cloud point can be measured quantitatively as the temperature at which the transmittance of a polymer solution goes below 50% of the initial value [37]. The study of the cloud point as function of temperature is shown in Figure 5.

From the definition of cloud point, we can see that in pure water the cloud point of PNIPAm is 33 °C, while the cloud point of the copolymers is 34 °C for PNIPAm-g-PMEP 95:5 and 35 °C for PNIPAm-g-PMEP 90:10. The behavior of chains made of pure PNIPAm is consistent with many studies in the literature, where similar values of cloud point were observed [37,38]. The detected increase in the cloud point for synthetized copolymers is due to the hydrophilicity of the phosphate side-chains. It is known that adding hydrophilic moieties can move the cloud point to higher temperatures, and vice versa for hydrophobic units [37]. Afterwards, the effect of salt concentration on the cloud point of the polymers was evaluated since the adhesion measurements were performed in sodium chloride solutions (see the Supplementary Materials). No differences in the cloud point could be seen when only a small amount of salt is added (10^−3^ M NaCl), while for a higher amount of salt (10^−1^ M NaCl) the cloud point was slightly shifted to lower temperatures. This is the so-called ‘salting out effect’ and it has been reported in previous studies [39].

### 2.4. Characterization of the Modified Surfaces

On the other hand, the LCST point of the thin polymer brush layer could differ from the one of the bulk material. In order to gain information about the LCST values as well as the swelling properties of the designed thin polymer brush layers with respect to their degree of functionalization, spectroscopic ellipsometry measurements were performed. The swelling ratio of the measured systems was defined as

(1) $$swelling\;ratio=\frac{thickness\;underwater}{dry\;thickness}$$

**Figure 6:**
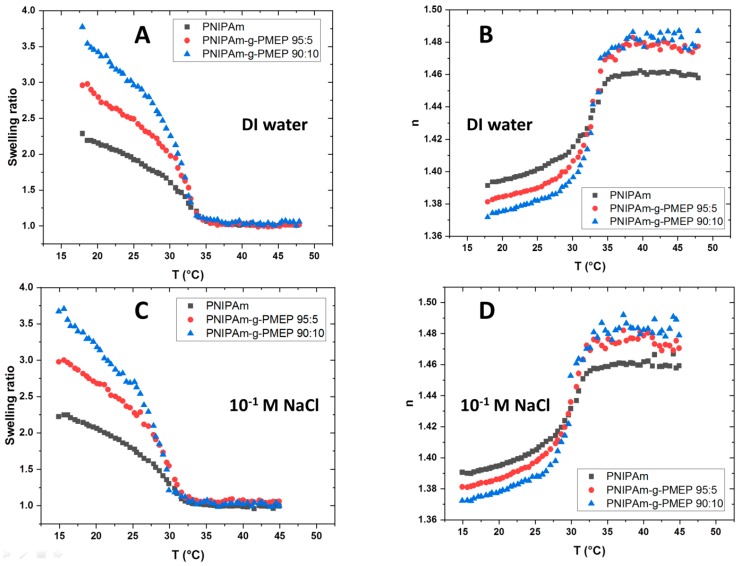
Swelling ratio and refractive index (n) in DI water (**A**,**B**) and in 10^−1^ M NaCl (**C**,**D**); black: PNIPAm; red: PNIPAm-g-PMEP 95:5; blue: PNIPAm-g-PMEP 90:10.

Above the LCST, the brushes are completely collapsed and therefore the swelling ratio will be close to 1, whereas at room temperature this value will increase. We defined the LCST as the temperature where the swelling ratio is in the middle between the maximum and minimal value. We measured the swelling ratio of thin polymer brush layers in the range of temperature from 20 °C to 50 °C and at salt concentration of 0 M and 0.1 M, as shown in Figure 6. The thickness of all systems both in dry and underwater conditions is presented in Table 3:

Figure 6A shows the swelling ratio in DI water, where the measured LCST is lower than the values found for the polymers in the bulk solution. When the polymer layer is grafted on a solid substrate, the LCST values are 30 °C for pure PNIPAm, 31 °C for PNIPAm-g-PMEP 95:5 and 31 °C for PNIPAm-g-PMEP 90:10. As expected, the presence of phosphate side-chains leads to a higher swelling behavior at room temperature due to their hydrophilicity. Despite these differences at room temperature, all layers collapse to a thickness of 30–35 nm at T > LCST, similar to the one in dry conditions. A resembling trend can be seen also when salt is added (Figure 6C). The LCST moves from 30 °C down to 28 °C for PNIPAm, from 31 °C to 29 °C for PNIPAm-g-PMEP 95:5 and from 31 °C to 29 °C for PNIPAm-g-PMEP 90:10. However, the swelling behavior at room temperature is not affected by the presence of salt. Moreover, it can be seen that the temperature-triggered transition is much sharper in the bulk than on the surface. This behavior comes from the fact that tethered brushes have more steric constrains compared to free chains in solution, and therefore the collapse of the layer takes more time, as observed by Fery and coworkers [40]. Another proof that the polymer layer is indeed collapsing at T > LCST can be obtained by studying the variation of the refractive index (n) according to the temperature. In general, the refractive index describes the packing density of a material: an increase of refractive index indicates the appearance of a denser material [41]. Figure 6B,D show that the refractive index of all layers increases when T approaches the LCST, and this is consistent with the decreased thickness of the film, because a more densely packed phase is forming due to the collapse of the chains and expulsion of water molecules from the layer (de-swelling). Our results are in good qualitative agreement with previous studies for the PNIPAm reference [42,43] while some differences can be found for the grafted architecture. Synytska and coworkers [25] compared the swelling of PNIPAm with graft P(OEGMA-MEO_2_MA) and P(OEGMA-OPGMA) polymer brushes and measured higher swelling for PNIPAm compared to the layers with graft architecture. They justified their findings considering the increasing stiffness of the side graft chain architecture due to the presence of side-chains. The discrepancy with our study can be attributed to the low amount of side-chains present in our graft polymer brush layers, which does not affect the overall stiffness of the polymer brushes. From the ellipsometric measurements, it can be seen that the thin PNIPAm-g-PMEP layers are able to change their properties between room temperature and the temperature of human body, which makes them suitable for biomedical applications.

**Figure 7:**
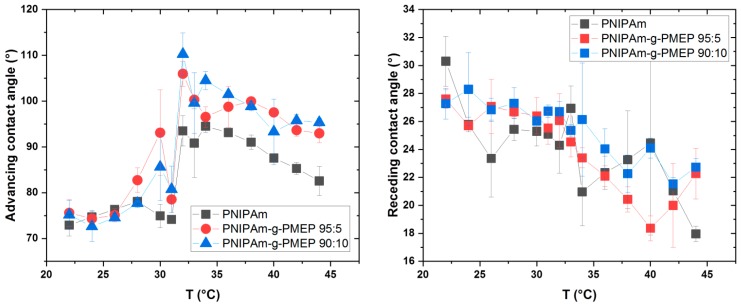
Advancing (**left**) and receding (**right**) contact angle measured by captive bubble technique (black: PNIPAm; red: PNIPAm-g-PMEP 95:5; blue: PNIPAm-g-PMEP 90:10).

Next, we studied the effect of the degree of functionalization in PNIPAm-g-PMEP brushes with side graft chain architecture on their wettability. The hydrophilic and/or hydrophobic properties of a surface can play a significant role in interfacial phenomena such as adhesion, and therefore the control over such properties can help the fine tuning of the adhesive behavior. We used the captive bubble technique instead of the usual sessile drop method because it allows measurements in liquid environment, therefore providing a more ‘realistic’ evaluation of the wettability of polymer brushes (e.g., no influence of the swelling due to the liquid drop). PNIPAm films are known to change their wettability when the LCST is reached: swollen and hydrophilic chains become collapsed and more hydrophobic [44], even if the debate about the effective hydrophobicity of PNIPAm above the LCST is still open [45]. The results for pure PNIPAm and for both copolymers are presented in Figure 7.

The advancing contact angle (Figure 7 left) at room temperature is 72° for PNIPAm and 75° for both copolymers, indicating that all our brushes are hydrophilic in these conditions. The contact angle then increases when the temperature starts to approach the LCST, coming to the value of 93° for PNIPAm, 105° for PNIPAm-g-PMEP 95:5, and 110° for PNIPAm-g-PMEP 90:10 when the LCST is reached; therefore, the polymer layers show hydrophobic behavior when T ≥ LCST. In addition, two interesting and unexpected behaviors can be observed for all measured samples. The first one is immediately before reaching the LCST. When the heating starts, the contact angle starts to increase (in the range between 25 °C and 30 °C) but a clear decrease can be seen at 28–32 °C, just before the jump to high values of advancing contact angle due to the complete collapse of the tethered chains. A similar behavior was observed by Leckband and coworkers [44], but no possible explanations were proposed. The second interesting behavior is the slight decrease in advancing contact angle at T > LCST. Immediately after the transition temperature, there is a jump in the contact angle to more hydrophobic values; but when the temperature is further increased all layers show a slight tendency to become more hydrophilic. A reasonable hypothesis could be that a rearrangement can take place at the surface of the brushes, but no reports of similar behavior have been found in the literature. A more detailed discussion of these features can be found in the Supplementary Materials. Figure 7 right shows the evolution of the receding contact angle as a function of temperature. The trend in this case is inversed compared to the advancing contact angle, since all layers show a decrease of receding contact angle when the temperature is increased. Also, the magnitude of this change is lower compared to the receding contact angle (around 10° for all brushes). The tendencies of both advancing and receding contact angles can be explained by the formation of hydrophobic domains when the temperature is approaching the LCST: the appearance of these domains due to the collapse of PNIPAm form a barrier for the motion of the water front, and therefore the advancing contact angle increases. Similarly, these same domains hold back the water front during the receding part, and this reflects in a decrease of the receding contact angle. Our results for PNIPAm brushes are in good qualitative agreement (except for the two features discussed earlier) with several previous studies on PNIPAm [44,46,47,48]. The discrepancies between measured values are mainly due to the different techniques used to measure the contact angle and to the different approaches in the preparation of the samples [48].

**Figure 8:**
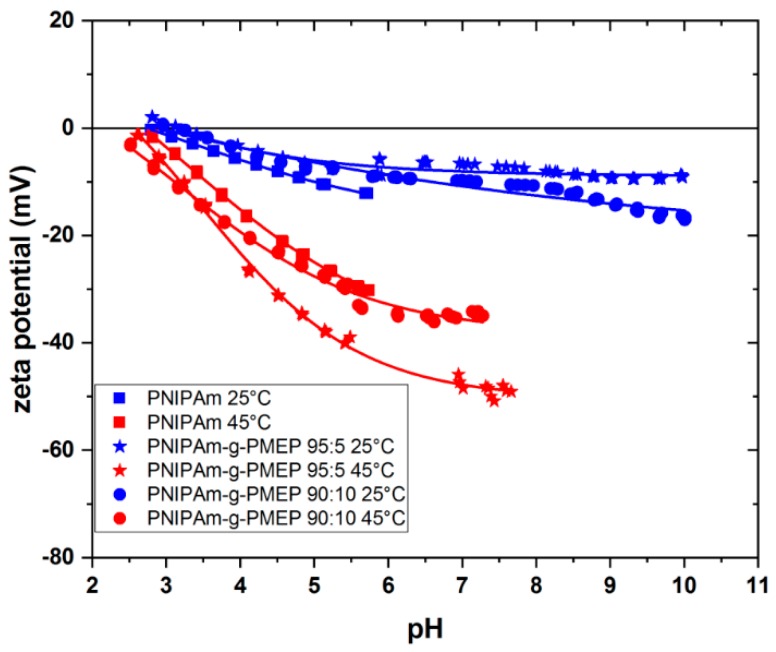
Apparent zeta potential values of PNIPAm (squares), PNIPAm-g-PMEP 95:5 (stars) and PNIPAm-g-PMEP 90:10 (circles) at T = 25 °C (blue) and T = 45 °C (red).

Next, we studied the surface charge of our layers by electrokinetic streaming potential measurements as shown in Figure 8.

The values of zeta potential were calculated from the Smoluchowski equation [49]:

(2) $$\zeta =\frac{dU}{dp}\frac{\eta }{\varepsilon r\cdot \varepsilon 0}k$$

where *U* is the streaming potential, *p* is the pressure loss, *εr* is the dielectric constant of the measuring solution (10^−3^ M KCl), *ε*0 is the vacuum permittivity, *η* is the viscosity and *k* is the conductivity of the measuring solution. In the case of solid surfaces, the zeta potential indicates the electrical potential at the shear plane. It is defined by the nature of the surface, its charge, the type of electrolyte, its concentration in the solution and by the solvent used [50]. As it is seen from the Figure 8, the zeta potential decreases while the pH increases and all layers show the isoelectric point (IEP) at pH ≈ 3. Since there are no ionizable groups in the thermo-responsive brushes, we suppose that this behavior is due to phenomena of ion adsorption; at pH < IEP, the surfaces show positive net charge due to the adsorption of H^+^ ions, while at pH > IEP anions such as OH^−^ or Cl^−^ are adsorbed and the net charge is negative. Moreover, polymer layers show more or less flat curve at 25 °C, and it is supposed that this behavior is due to the strong swelling of the brushes, with small contribution from the ion adsorption. On the contrary, the behavior at T > LCST is different: the absolute value of the zeta potential increases because a more collapsed and hydrophobic surface promotes the adsorption of ions. It can be observed that all investigated brushes show the same behavior and the same switching when T > LCST. A previous study from our group showed the same trend for pure PNIPAm brushes [25].

### 2.5. Underwater Adherence between Native SiO_2_ Colloidal Probe and Graft Polymer Brushes

**Figure 9:**
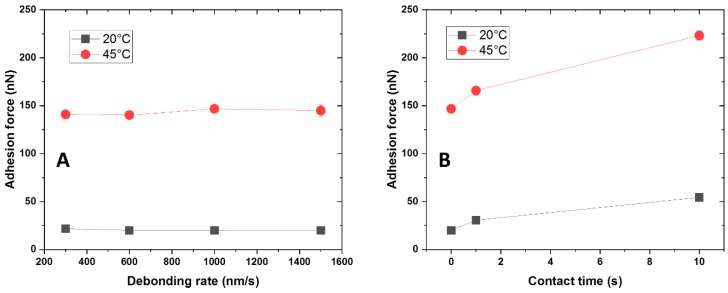
Influence of debonding rate (**A**) and contact time (**B**) on the pull-off force for PNIPAm-g-PMEP 90:10 against a SiO_2_ CP (all measurements were carried out in 10^−3^ M NaCl); black: 20 °C; red: 45 °C.

Atomic force microscopy (AFM) was developed during the 1980s in order to measure ultra-small forces on very small particles, even single atoms [51]. The setup was initially developed for imaging and topography of surfaces, but in the last few decades this technique has been more and more applied to the measurement of intermolecular forces in air and in liquid environment. The colloidal probe (CP) approach was introduced in 1991 [52,53]. Many advantages can be obtained by using smooth spherical particles with defined radius instead of a normal cantilever tip. The force evaluation can be done quantitatively, while enlarging the interaction cross section increases the total measured force avoiding resolution limitations for weak interactions on soft materials. Additionally, these spherical particles can be chemically modified, allowing the measurement against a large number of probes with different chemistry and composition. AFM-CP force measurements were performed to evaluate the adhesion force between PNIPAm-g-PMEP with side graft chains and colloidal probes modified with polar and apolar silanes. If adhesion is involved, the retraction part of the curve shows a minimum which corresponds to the pull-off force, which is defined as the maximum force applied before adhesive failure. This is why the pull-off force is commonly used to describe surface–probe interactions [54]. To study the adhesive behavior of the brushes with side graft chains, we investigated the influence of two parameters on the pull-off force, debonding rate, and contact time, using a SiO_2_ colloidal probe (diameter ≈ 20 µm); representative raw curves can be found in the Supplementary Materials. Firstly, we studied the influence of the debonding rate at constant load (100 nN) and without dwelling of the probe against the substrate. The results are presented in Figure 9A.

**Figure 10:**
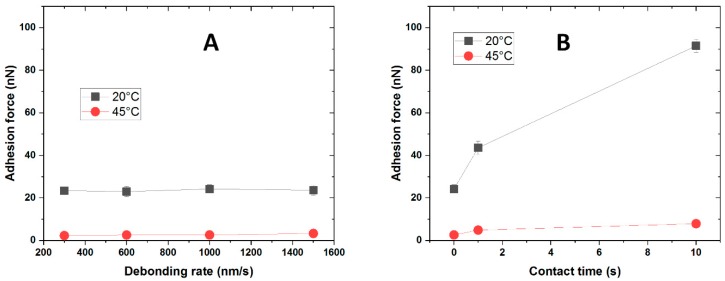
Influence of debonding rate (**A**) and contact time (**B**) on the pull-off force for PNIPAm-g-PMEP 90:10 against 1H,1H,2H,2H-perfluorooctyldimethylchlorosilane CP (all measurements were carried out in 10^−3^ M NaCl); black: 20 °C; red: 45 °C.

We systematically varied the debonding rate between 300 nm/s and 1500 nm/s both at T < LCST and at T > LCST. At both temperatures, the pull-off force does not show any variation when the debonding rate is increased. A dramatic increase of adhesive properties can be observed when the temperature is raised above the LCST of the copolymer, but the velocity at which the probe is retracted does not affect its general behavior. We therefore chose 1000 nm/s as operating condition. Afterwards, the effect of the contact time was studied (Figure 9B), keeping 1000 nm/s as debonding rate and 100 nN as applied load. At room temperature, an increase of pull-off force was observed when the contact time between surfaces is increased from 0 to 1 s: the pull-off force goes from 19 nN to 30 nN, resulting in an increase of almost 60% in adhesion. When the contact time is prolonged to 10 s, a further increase of 24 nN is observed, meaning that going from ‘zero’ contact time to 10 s, the pull-off force increases of around 200% of the initial value. This substantial improvement of adhesive properties can be explained by considering that at T < LCST the brushes are swollen and soft, therefore being able to increase the contact area when the two surfaces are maintained against each other for a certain amount of time. At T > LCST the same trend was observed. The pull-off force goes from 146 nN at ‘zero’ contact time to 166 nN after 1 s, resulting in a variation of around 15%; when the two surfaces are allowed to stay in contact for 10 s, the adhesion is further increased to 223 nN, corresponding to an increase of 50%. In these conditions, the brushes are collapsed and stiffer, therefore the increase of contact area due to longer contact between surfaces is lower compared to the same situation at T < LCST. The most known models for adhesion predict no influence of the contact time on the pull-off force. However, this assumption is valid only when the deformations are purely elastic [55]. This suggests that the deformations in our system can have a visco-elastic component; since the SiO_2_ CP are considered as purely elastic solids, we make the hypothesis that the visco-elastic behavior can be attributed to the responsive polymer layer. The hydrophilic silica probe was then modified with 1H,1H,2H,2H-perfluorooctyldimethylchlorosilane in order to increase its hydrophobicity. The effect of debonding rate and contact time was studied again as shown in Figure 10, while representative raw data can be found in the Supplementary Materials.

No influence of the debonding rate was observed, similarly to the measurements using hydrophilic silica CP. Interestingly, the switching is inversed compared to the previous situation: high adhesion was measured at low temperature, while only very low adhesion was detected above the LCST. As it was seen for the silica probe, also in this case a clear effect of the contact time on the adhesive properties was observed: adhesion increases when the two surfaces are kept in contact for a longer time. The effect is extremely pronounced at low temperature, where an increase in adhesion of almost 300% when the contact time goes from ‘zero’ to 10 s. On the contrary, the magnitude of this phenomenon decreases when the temperature is raised above the LCST.

**Figure 11:**
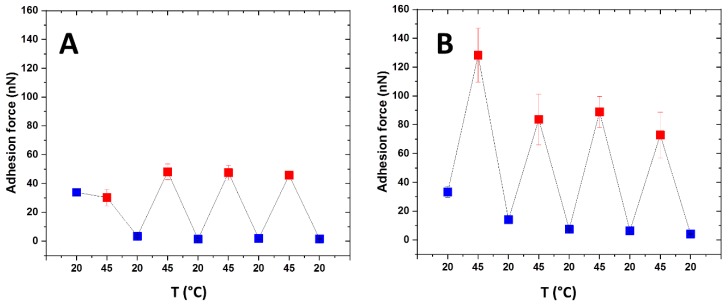
Adhesion cycles for PNIPAm-g-PMEP 95:5 (**A**) and PNIPAm-g-PMEP 90:10 (**B**) (all measurements were carried out in 10^−3^ M NaCl); blue: 20 °C; red: 45 °C.

Afterwards, we studied the reversibility of the switching behavior by performing adhesion cycles against an unmodified SiO_2_ probe. Adhesion was measured at room temperature, then the temperature was increased to 45 °C and the adhesion was measured again after equilibration; the system was then cooled down again to room temperature for a new measurement and so on for a total of four cycles 20 °C/45 °C. We studied both copolymers to ensure that all systems show reversible behavior. The debonding rate was kept at 1000 nm/s, with a contact time of 0 sand with an applied load of 100 nN. The results are shown in Figure 11.

For PNIPAm-g-PMEP 95:5 (Figure 11A), the first measurement provides unexpected high adhesion at room temperature, even higher than the first measurement at T > LCST; in the following cycles, the adhesion forced is stabilized both below and above the LCST, resulting in an adhesion force of 2 nN at 20 °C and 47 nN at 45 °C. For PNIPAm-g-PMEP 90:10 (Figure 11B) we also observed a deviation between the first cycle, where both at 20 °C and 45 °C higher adhesion was measured, and the remaining ones where the pull-off force stabilized to 7 nN at 20 °C and 80 nN at 45 °C. From these measurements, we concluded that indeed the polymer layers are able to switch their adhesive properties, and also that the switching can be reversibly observed over several heating/cooling cycles, although an equilibration of the system can take place during the first cycle of heating and cooling.

### 2.6. Underwater Adhesion against Different Substrates

**Figure 12:**
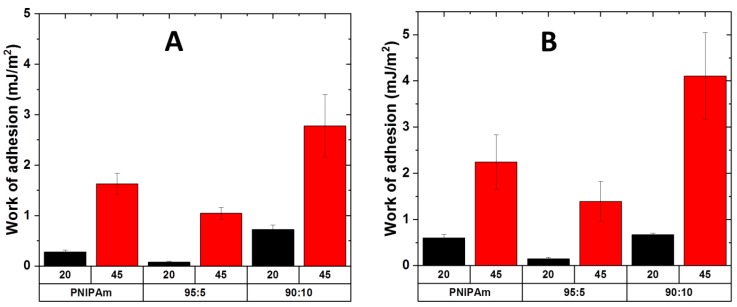
Underwater work of adhesion against a native SiO_2_ probe in 10^−3^ M NaCl (**A**) and in 10^−1^ M NaCl (**B**); black: 20 °C; red: 45 °C.

The pull-off force is a good indicator of the adhesive properties of surfaces, but many experimental parameters can have an influence on the measured force. For this reason and for easier comparisons between different systems, it is more convenient to calculate the work of adhesion (w), which is the energy necessary to create two surfaces. To derive the adhesion energy, an adequate contact mechanic theory must be used to treat the raw data. The first model was proposed by Hertz to describe the contact between elastic bodies, but without taking adhesion phenomena into account [56] (English version [57]). Several theories were then proposed to take into account the adhesion contributions; the two most common models have been proposed by Johnson, Kendall, and Roberts (JKR model) [58] and by Derjaguin, Muller, and Toporov (DMT model) [59]. Tabor [60] proposed a parameter (µ_T_) that allows choosing between the two models based on the deformation taking place and on the magnitude of the surfaces’ forces.

(3) $${µ}_{T}\;=\;\left(\frac{16\;R\;w^{2}}{9\;K^{2}\;{z_{0}}^{3}}\right)1/3$$

where *R* is the effective radius, *w* is the work of adhesion, *K* is the effective elastic modulus, and *z*_0_ is the interatomic equilibrium distance for solid–solid interactions in the Lennard-Jones potential (usual values are 0.3 nm to 0.5 nm). *R* and *K* can be calculated by

(4) $$\frac{1}{R}\;=\;\frac{1}{R1}+\frac{1}{R2}$$

(5) $$\frac{1}{K}=\frac{3}{4}\left(\frac{1-{\nu 1}^{2}}{E1}+\frac{1-{\nu 2}^{2}}{E2}\right)$$

where *R1* and *R2* are the radii of the two surfaces, *E1* and *E2* are their Young moduli, and *ν*1 and *ν*2 are their Poisson ratios. For µ_T_ > 5 the JKR model is valid while for µ_T_ < 5 the DMT model is valid. In our study, we assumed *E1* = 10 GPa and *ν*1 = 0.5 for the silica particles used as colloidal probe, *E2* = 100 kPa and *ν*1 = 0.5 for the polymer layer, a work of adhesion of *w* = 0.1 mJ/m^2^ and *z*_0_ = 0.5 nm. We are describing a micrometric particle (*R1* ≈ 10 µm) making contact with a planar surface (*R2* → ∞), therefore *R* = *R1*. Our calculations resulted in a Tabor parameter µ_T_ > 30, therefore the JKR model was chosen. This model describes the contact area between two bodies as a function of the adhesion energy of the applied load and of the elastic properties of the two surfaces. The work of adhesion can be calculated from the pull-off force by

(6) $$w\;=\;\frac{2}{3\pi R_{\mathrm{CP}}}F$$

where RCP is the radius of the colloidal probe (RCP = 9795 µm) and F is the pull-off force. All results shown in this part have been obtained using this approach; representative raw curves can be found in the Supplementary Materials. Firstly, we studied the adhesive properties of our layers below and above the LCST against a SiO_2_ probe at different conditions of salt concentration. The results are presented in Figure 12.

At low salt concentration (Figure 12A) all systems show responsive adhesive behavior. The adhesion decreases in the sequence PNIPAM-g-PMEP 90:10, PNIPAm and PNIPAm-g-PMEP 95:5. Moreover, PNIPAM-g-PMEP 90:10 is able to retain the thermal switching of PNIPAm (the ratio between adhesion at 45 °C and at 20 °C is similar, 4.2 for PNIPAm-g-PMEP 90:10 and 4.6 for PNIPAm) while improving the maximum value of adhesion energy (2.8 mJ/m^2^ compared to 1.6 mJ/m^2^). Increasing the ionic strength (Figure 12B) has no influence on the general tendency, as expected for non-ionizable chains where electrostatic interactions have no role in the adhesion phenomena, and it seems to even improve the overall adhesive properties. The analysis of the raw curves (see Supplementary Materials) reveals that in all conditions the indentation at 20 °C is higher than the indentation at 45 °C, which is consistent with the transition between swollen and collapsed layer when the LCST is reached. In addition, the shape of the retraction part of the curves confirms this hypothesis: a smoother and broader rupture event can be seen in the curves at 20 °C, corresponding to the separation between the probe and swollen brushes, while the rupture at 45 °C is sharper, corresponding to the separation between the probe and a collapsed film. The shape of the retraction curve at 20 °C indicates a continuous detachment, as previously observed in several polymer systems [61]; at 45 °C, the shape indicates a single rupture event with the so-called jump off contact. Similar behaviors have been observed for PNIPAm and other polar thermo-responsive layers [25,30,40,46]. We make the hypothesis that, at room temperature, the brushes are swollen and hydrophilic, and adhesion against hydrophilic surfaces comes from weak hydrophilic interactions such as H-bonds. When the LCST is reached, the layer collapses and therefore the density of functionalities at the surface able to establish H-bonds with the probe increases. Also, the presence and the amount of polar side-chains play a role. For the copolymer, 95:5 the amount of methylphopshate is low and it gives H-bonding mainly with PNIPAm units, therefore subtracting some interactions between probe and substrate. When the content of phosphate increases, the number of interactions between side-chains and probe increases as well, a higher number of interactions is established and adhesion is higher compared to pure PNIPAm.

**Figure 13:**
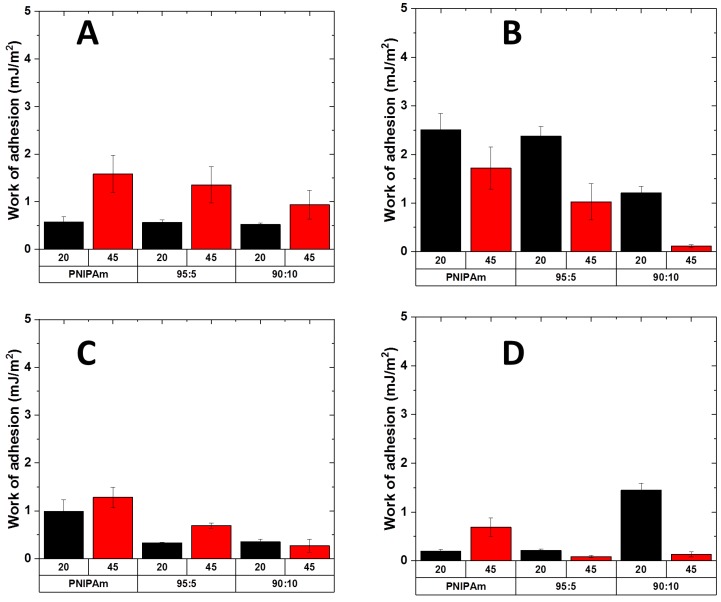
Work of adhesion against hydrophilic amino probe (**A**) 10^−3^ M NaCl; (**C**) 10^−1^ M NaCl) and hydrophobic fluoro probe (**B)** 10^−3^ M NaCl; (**D)** 10^−1^ M NaCl); black: 20 °C; red: 45 °C.

Secondly, we chemically modified the surface of the colloidal probe in order to study the adhesion of the polymer brushes against different substrates. SiO_2_ probes (contact angle: 20°) were converted to hydrophilic positively charged probes (covered with 3-Aminopropyltriethoxysilane, contact angle: 65°) and hydrophobic probes (covered with 1H,1H,2H,2H-Perfluoro- octyldimethylchlorosilane, advancing contact angle > 100°). The results are presented in Figure 13.

The measurements against amino modified probes resulted in similar adhesion and switching behavior for pure PNIPAm and for the copolymer PNIPAm-g-PMEP 95:5, while the copolymer PNIPAm-g-PMEP 90:10 seems to lower its performances compared to SiO_2_ probe. At low salt concentration (Figure 13A) the overall trend is similar to the measurements with silica probe, yielding higher adhesion at T > LCST even if the general switching behavior is less prominent. Increasing the salt concentration (Figure 13B) results in a decrease in performances: adhesion above the LCST decreases and therefore the responsive behavior of all layers is diminished. Surprising results were obtained with the fluoro probes as well. The behavior of PNIPAm is different compared to the copolymers: pure PNIPAm shows slightly higher adhesion at room temperature than at high temperature at low salt concentration (Figure 13C) and the opposite trend at higher ionic strength (Figure 13D). However, the switching ability is significantly lower than the measurements with SiO_2_ probes. Interestingly, both copolymers show inversed trend compared to the previous measurements: higher adhesion is measured at room temperature, while at T > LCST the grafted brushes are poorly adhesive against hydrophobic substrates. We suppose that the adhesion at room temperature arises from the hydrophobic interactions between the probe and the hydrophobic methyl groups of PNIPAm units; at T > LCST, these units form intra-chain bonding and therefore they are not available anymore for interactions with the substrate; moreover, the presence of hydrophilic side-chains contribute to decrease the adhesion since they cannot interact with the hydrophobic probe. Also, in these conditions, PNIPAm-g-PMEP 90:10 shows the most interesting behavior: even if the tendency is inversed, this sample shows dramatic switching of properties both against hydrophilic (native SiO_2_) and hydrophobic probes (1H,1H,2H,2H-Perfluoro- octyldimethylchlorosilane). Furthermore, the general tendency of this copolymer against both silica and hydrophobic probe is not affected by the presence of salt. Thus, this seems to be the most suitable architecture and composition for achieving reversible thermo-responsive adhesion in underwater conditions against both hydrophobic and hydrophilic substrates.

## 3. Materials and Methods

### 3.1. Materials

Ammonia solution (NH_4_OH, Acros, 28–30% solution, Thermo Fisher Scientific, New Jersey, US), hydrogen peroxide (H_2_O_2_, VWR, 30%, Radnor, PA, USA), ethanol abs. (EtOH, VWR, 99.9%, Radnor, PA, USA), 3-aminopropyl- triethoxysilane (APTES, ABCR, 97%, Karlsruhe, Germany), a-bromoisobutyryl bromide (BrIn, Aldrich, 98%, Merck Group, St. Louis, MS, USA), hydrochloric acid (Merck, 37%, Merck Group, Darmstadt, Germany), potassium hydroxide (Fixanal, 99%, Honeywell, Charlotte, NC, USA), *N*,*N*-Dimethylformamide (Aldrich, 99.8%, Merck Group, St. Louis, MS, USA), anhydrous dichloromethane (Fluka, Honeywell, Charlotte, NC, USA), n-hexane (Merck, 99%, Merck Group, Darmstadt, Germany), triethylamine (Fluka, 99.5%, Honeywell, Charlotte, North CA, USA), copper(II) bromide (CuBr_2_, Aldrich, 99.999%, Merck Group, St. Louis, MS, USA), *N*,*N*,*N′*,*N″*,*N″*-pentamethyldiethylenetriamine (PMDTA, Aldrich, 99%, Merck Group, St. Louis, MS, USA), ethyl a-bromoisobutyrate (EBiB, Aldrich, 98%, Merck Group, St. Louis, Missouri, US), l-ascorbic acid (Aldrich, 99%, Merck Group, St. Louis, MS, USA), potassium chloride (Aldrich, 99.8%, Merck Group, St. Louis, MS, USA), and sodium chloride (Aldrich, 99.8%, Merck Group, St. Louis, MS, USA) were used as received. *N*-Isopropylacrylamide (NIPAm, Aldrich, 97%, Merck Group, St. Louis, MS, USA) was recrystallized in hot hexane prior to use. Polished single-crystal silicon wafers of (100) orientation (Si-Mat Silicon Materials, Landsberg, Germany) were used as solid substrates. Millipore water was obtained from Milli-Q setup (Millipore, conductivity: 0.055 µS/cm). Diazabicycloundecene (DBU, Aldrich, 98%, Merck Group, St. Louis, MS, USA), 2-(benzyloxy)ethanol (Aldrich, 98%, Merck Group, St. Louis, MS, USA), and triethylamine (Roth, dried with CaH_2_, Karlsruhe, Germany) were distilled and stored over molecular sieves (4 Å). 2-chloro-2-oxo-1,3,2-dioxaphospholane (COP, Aldrich, 95%, Merck Group, St. Louis, MS, USA), dichloromethane (DCM, Acros Organics, dry, 99.9%, Thermo Fisher Scientific, New Jersey, US), dichloromethane (DCM, Fisher scientific, p.a., Thermo Fisher Scientific, NJ, USA), diethylether (Aldrich, p.a., Merck Group, St. Louis, MS, USA), ethylacetate (Fisher scientific, p.a., Thermo Fisher Scientific, NJ, USA), methanol (Aldrich, anhydrous, 99.8%, Merck Group, St. Louis, MS, USA), tetrahydrofuran (THF, Acros Organics, dry, 99.85%, Thermo Fisher Scientific, NJ, USA), 2-Isocyanatoethyl methacrylate (TCI, >98.0%, Tokyo, Japan), hydroquinone (Aldrich, ≥99%, Merck Group, St. Louis, MS, USA) were used without further purification.

### 3.2. Synthesis of Poly(Methyl Ethylene Phosphate) Methacrylate (PMEP)

#### 3.2.1. 2-Methoxy-2-oxo-1,3,2-dioxaphospholane (MEP)

A flame-dried 1000 mL three-neck flask, equipped with a dropping funnel, was charged with 2 chloro-2-oxo-1,3,2-dioxaphospholane (COP) (50 g, 0.35 mol) dissolved in dry THF (300 mL). A solution of dry methanol (11.24 g, 0.35 mol) and dry trimethylamine (35.42 g, 0.35 mol) in dry THF (45 mL) was added dropwise to the stirring solution of COP at −20 °C under argon atmosphere. During reaction, hydrogen chloride was formed and precipitated as triethylamine hydrochloride. The reaction was stirred at 4 °C overnight. The salt was removed by filtration and the filtrate concentrated in vacuo. The residue was purified by distillation under reduced pressure to give a fraction at 89–97 °C at 0.001 mbar, obtaining the clear, colorless, liquid product MEP (37.21 g, 0.27 mol, yield: 77%). ^1^H-NMR (500 MHz, DMSO-d_6_): δ 4.43 (m, 4H, O-CH_2_-CH_2_-O), 3.71 (d, 3H, O-CH_3_). ^13^C{H} NMR (125 MHz, DMSO-d_6_): δ 66.57 (s, 2C, O-CH_2_-CH_2_-O), 54.72 (s, 1C, O-CH_3_). ^31^P{H} NMR (202 MHz, DMSO-d_6_): δ 17.89.

#### 3.2.2. Polymerization to PMEP

*N*-cyclohexyl-*N*′-(3,5-bis(trifluoromethyl)phenyl)thiourea (TU) was synthesized according to the procedure in [62]. TU and the initiator 2-(Benzyl)oxyethanol were freeze dried with benzene prior use. MEP (14.0 g, 10.1·10^−2^ mol), TU (6.26 g, 16.9·10^−3^ mol), 2-(Benzyl)oxyethanol(0.55 g, 1.37·10^−3^ mol) and 23.56 mL dry dichloromethane were introduced into a 100 mL flame dried Schlenk tube to give a total reaction concentration of 4 mol/L MEP in dichloromethane. The mixture was cooled down to 0 °C and polymerization was initiated by rapid addition of 2.52 mL 1,8-Diazabicyclo[5.4.0]undec-7-ene (DBU) (2.57 g, 16.90·10^−3^ mol) to the stirring solution with a syringe. The polymerization was quenched after 1 h by excess addition of 2-Isocyanatoethyl methacrylate (2.62 g, 16.90·10^−3^ mol). The polymer was precipitated twice from dichloromethane into ice-cold ethyl acetate and once into ice-cold diethyl ether. 0.22 wt% hydroquinone was added to the polymer, the mixture dissolved in deionized water and then freeze-dried. The polymer was obtained after freeze drying as a viscous colorless material in a yield of 84%. ^1^H NMR (300 MHz, DMSO-d_6_) δ 8.16–6.71 (m, 9H), 4.44 (s, 2H), 4.29–3.93 (m, 132H), 3.78–3.53 (m, 104H). ^13^C{H} NMR (75 MHz, Chloroform-d) δ 66.51, 54.81, 54.73. ^31^P{H} NMR (121 MHz, DMSO-d_6_) δ 1.10, −0.12, −1.28. ^1^H NMR (300 MHz, DMSO-d_6_) δ 7.42–7.23 (m, 5H, aromatic CH), 6.55 (s, 0.25 H, Hydroquinone CH) 6.06 (s, 1H, -CH=CH_2_), 5.68 (s, 1H, -CH=CH_2_), 4.52 (s, 2H, benzyl -CH_2_-), 4.18 (h, J = 5.2 Hz, 123H, backbone –CH_2_-CH_2_-), 3.73 (s, 92H, -O-CH_3_), 1.87 (s, 3H, -C-CH_3_). ^31^P NMR (121 MHz, DMSO-d_6_) δ 1.11, −0.12, −1.32.

### 3.3. Synthesis of Grafted Brushes PNIPAm-g-PMEP

Polished single-crystal silicon wafers were chosen as solid substrate. In order to produce polymer brushes on the substrate, a suitable initiator for polymerization had to be placed on top of the wafers. To do so, three steps were necessary: (1) cleaning of the wafers (10 × 20 mm) with a mixture of NH_4_OH/H_2_O_2_/H_2_O (1:1:1), resulting in a uniform SiO_2_ layer carrying silanol groups (thickness: 1.4 ± 0.1 nm determined by null ellipsometry); (2) modification of the layer using a 3% solution of APTES in ethanol abs. (thickness: 0.7 ± 0.1 nm); and (3) modification of the layer using a BrIn solution (3% of trimethylamine and 1.5% of BrIn in anhydrous dichloromethane) (thickness: 0.5 ± 0.1 nm). Once the modification was done, the brushes were produced by ‘grafting through’ approach using ARGET-ATRP: 2 g of NIPAm and the corresponding amount of PMEP were added into a reaction tube containing two initiator-modified wafers, 3 mL of ethanol abs., 32 µL of CuBr_2_ (0.1 M in DMF), 32 µL of PMDTA (0.5 M in DMF), and 0.15 µL of EBiB. The tube was sealed and the mixture was degassed for 10 min using argon. Then 100 µL of L-ascorbic acid (1 M solution in DMF) were added, and the polymerization was carried out at 70 °C for 150 min. The wafers were then thoroughly washed with ethanol abs. and dried under a gentle flux of nitrogen. The bulk polymer was collected by precipitation and purified by dialysis (one week in DI water, cut-off: 10 kDa) prior to characterization.

### 3.4. Null Ellipsometry

The thickness of polymer brushes on silicon wafers in dry state was measured at λ = 632.8 nm and a 70° angle of incidence with a null-ellipsometer (Multiscope, Optrel Berlin, Wattwill, Switzerland) in a polarizer–compensator–sample–analyzer configuration.

### 3.5. In Situ Spectroscopic Ellipsometry

In situ ellipsometric measurements were performed to examine the swelling behavior of the polymer brushes at different temperatures using a precision cell for measurements in liquid environment (quartz glass, Hellma Analytics). A rotating compensator ellipsometer at multiple wavelengths was used (370–900 nm, alpha-SE, J.A. Woollam, Co. Inc., Lincoln, NE, USA). The sample was immersed in the measuring liquid (≈ 4 mL) and 30 min were given for equilibration purposes. The temperature-dependent measurements were carried out using an angle of incidence of 70° and a heating rate of 0.5 °C/min. For modeling, a two-layer system (silicon-silicon oxide/polymer brush, plus water as ambient) was assumed.

### 3.6. Dynamic Light Scattering

Zetasizer Nano ZS by Malvern Instruments, Spectris, Egham, UK was used for the determination of the hydrodynamic radius (R_H_) in polymer solutions (1 mg/mL). The device was equipped with a 633 nm laser and with a non-invasive back scatter technology. Every sample was measured three times, and the average value was used for the calculations.

### 3.7. Gel Permeation Chromatography

The molecular weight of polymers obtained from the bulk solution after precipitation and dialysis was determined using GPC (Gradient HPLC HP Series 1100, Agilent Technologies Inc., Santa Clara, CA, USA).

### 3.8. Turbidity Measurements via UV–Vis Spectroscopy

The cloud point measurements were performed with an aqueous polymer solution (5 mg/mL) using a UV–Vis spectrometer equipped with a temperature controller system (Agilent Technologies Inc., Santa Clara, CA, USA). The UV–Vis transmission properties of the solutions were studied in the temperature range of 20 to 50 °C to determine the precise cloud point. The measurements were carried out using a Varian Cary 50 spectrophotometer from Agilent Technologies (Agilent Technologies Inc., Santa Clara, CA, USA). The values of transmittance were read at λ = 563 nm.

### 3.9. Wettability via Captive Bubble Method

The polymer-modified wafers were fixed horizontally on a holder and immersed in the measuring solution (NaCl 10^−3^ M). After reaching the desired temperature, the system was allowed to equilibrate for at least 10 min. Measurements were carried out between 22 °C and 44 °C, with 2 °C increments. Between 30 °C and 34 °C, the temperature was increased by 1 °C. For each temperature, three air bubbles were created and withdrawn. The drop profiles were imaged using a CCD camera. Receding and advancing contact angles for the aqueous solutions were obtained by calculating the difference of the measured contact angle of the air bubble to 180°.

### 3.10. Streaming Potential Measurements

Electrokinetic measurements on polymer-modified wafers were performed as streaming potential experiments with an electrokinetic analyzer (EKA) by Anton Paar GmbH (Graz, Austria). A pair of wafers was mounted in a custom-built rectangular cell where the two surfaces form a thin streaming channel. pH-dependent measurements were carried out by adjusting the pH of the measuring solution (KCl 10^−3^ M) with KOH 10^−1^ M or HCl 10^−1^ M. The apparent electrokinetic potential (zeta potential) values were calculated from the measured streaming potential values according to Smoluchowski’s equation [49].

### 3.11. AFM-CP Force Measurements

For cantilever calibration and force measurements an MFP-3D AFM (Asylum Research Inc., Goleta, CA, USA), equipped with a BioHeater fluid cell with heating element (Asylum Research Inc.) was used. Cantilevers of series NSC36 (tipless, no aluminium coating, MikroMasch, NanoWorld AG, Schaffhausen, Switzerland) were used. For calibration of the cantilevers, the inverted optical lever sensitivity (invOLS, which is the proportionality constant between photodiode signal and cantilever deflection) was determined by performing at least nine force measurements against the hard surface of a cleaned microscopy glass slide, and fitting the linear part (repulsive part) of the force–distance curve. Afterwards, the thermal noise method was used to determine the spring constant [63]: all cantilevers had a spring constant between 0.7 N/m and 1.8 N/m. Using a micromanipulator on an optical microscope and glass capillary, a small droplet of two component epoxy glue (UHU plus endfest 300, UHU GmbH, Germany) was deployed on the cantilever, and using a second glass capillary a colloidal probe was placed on top of the glue, taking care not to turn it in order to leave the surface free from glue pointing away from the cantilever. After hardening of the glue, cantilevers with probes were briefly submerged in DI water and ethanol, to clean the colloid surfaces and confirm sufficient attachment. The back sides of the wafers were glued to round glass slides compatible with the fluid cell using two component epoxy resin. After glue hardening, two scratches forming a cross shape were made across the polymer brushes with the tip of cleaned tweezers, to allow access to uncovered wafer as reference surface and for orientation on the otherwise homogeneous sample surface during measurement. After immersion in the measuring solution, the sample and cantilever were given at least 30 min time for equilibration purposes. InvOLS was determined again performing at least nine measurements in three spots in the scratch of the sample, where the hard surface of the silicon wafer was exposed. The maximum force to be applied on the sample upon approach was set to 100 nN. Four to six force maps (20μm × 20μm), each consisting of 16 single force measurements were made in a box pattern around the crossing of the scratches on the surface. Care was taken to ensure sufficient distance to the scratches to measure on undisturbed polymer brush. After measuring at room temperature, the heating element was activated, and after the solvent reached its target temperature, another 30 min were given for equilibration purposes. Measurement of invOLS and force maps were repeated in the same manner, in approximately the same spots on the sample. Ten representative curves from each measurement were selected for further evaluation of the adhesive properties.

### 3.12. Chemical Modification of the Colloidal Probes

Native Silica CPs (diameter: 19.59 µm) were purchased from microParticles GmbH, Germany. Fluorosilane and aminosilane probes were prepared by functionalization of native silica CPs in gas phase. After oxidation in oxygen plasma the cantilevers were kept in a desiccator with 1H,1H,2H,2H-Perfluorooctyldimethylchlorosilane (Aldrich, 98%) or 3-Aminopropyltriethoxysilane for vapor deposition in reduced pressure for four hours, then cleaned thoroughly with ethanol abs. and DI water.

## 4. Conclusions

In summary, the synthesis of grafted thermo-responsive PNIPAm-g-PMEP brushes using ARGET-ATRP is presented. The study of the grafting density of the layers confirms that the thin films are in the brush regime. The responsive behavior of the final layers was characterized studying the surface-grafted polymer as well as the polymer from the bulk solution, and pure PNIPAm brush was used as reference. PNIPAm-g-PMEP copolymers retain the responsive behavior of PNIPAm: when T > LCST, a clear switching of properties is observed. More specifically, all layers above the critical temperature show collapse of the chains, increased hydrophobicity, and variation of the surface charge even if no ionizable groups are present. Secondly, effect of adhesion parameters—such as debonding rate and contact time—was studied. Results indicate that there is no influence of the debonding rate on the adhesion, whereas increasing the time of contact between surfaces improves the adhesion. Thirdly, the reversibility of the adhesive properties was confirmed by performing adhesion cycles. Finally, the adhesive properties of the layers are studied below and above the LCST against hydrophilic and hydrophobic substrates. All layers show responsive behavior against silica probes, while performances are lower against a hydrophilic aminosilane probe. Interestingly, the adhesion trend is inversed against hydrophobic substrates, where the grafted layers are highly adhesive at room temperature and almost completely not adhesive above the LCST. Moreover, we show that a higher ratio of polar side-chains in the brushes correspond to improved switching behavior during the adhesion measurements, both against hydrophilic and hydrophobic substrates. The findings presented here could be useful in the design of responsive thin films able to switch their adhesive properties underwater against a plethora of different surfaces.

## Figures and Tables

**Table 1:** Surface modification on silicon wafers and corresponding thickness.

Layer	Thickness (nm)
SiO_2_	1.4 ± 0.2
3-aminopropyl- triethoxysilane	0.7 ± 0.1
α-bromoisobutyryl bromide	0.5 ± 0.1

**Table 2:** Values of grafting density (σ), distance between grafting points (D), molecular weight (Mn), and dry thickness of PNIPAm and graft PNIPAm-g-PMEP polymer brushes.

Ratio Pnipam:Phosphate Units	σ (Chains/nm^2^)	D (nm)	Mn (kDa)	Dry Thickness (nm)
100:0	0.22	2.1	83	30.4 ± 0.2
95:5	0.26	1.8	55	35.1 ± 0.7
90:10	0.23	2.0	69	32.9 ± 0.9

**Table 3:** Thickness in dry conditions and underwater (DI water, 20 °C) for all thin polymer brush layers.

Ratio PNIPAm:Phosphate Units	Dry Thickness (nm)	Underwater Thickness at 20 °C (nm)
100:0	30.4 ± 0.2	69.5 ± 1.8
95:5	35.1 ± 0.7	104.8 ± 3.5
90:10	32.9 ± 0.9	120.7 ± 4.1

## Supplementary Materials

Supplementary Materials can be found at https://www.mdpi.com/1422-0067/20/24/6295/s1. Content: NMR spectra of PNIPAm-g-PMEp 95:5 and 90.10; Detailed discussion concerning the grafting density (σ); Influence of salt concentration on the cloud point of bulk polymer brushes; representative curves for AFM-CP against SiO2 CP—Debonding rate and contact time; Representative curves for AFM-CP against 1H,1H,2H,2H-Perfluorooctyldimethylchlorosilane CP—Debonding rate and contact time; Representative curves for AFM-CP against SiO_2_ CP—Influence of salt concentration; Representative curves for AFM-CP against 3-Aminopropyltriethoxysilane CP—Influence of salt concentration; Representative curves for AFM-CP against 1H,1H,2H,2H-Perfluorooctyldimethylchlorosilane CP—Influence of salt concentration; Analysis of variation of contact angle using PNIPAm-PMEP 95:5 as example.

## Abbreviations

PNIPAm	Poly(*N*-isopropylacrylamide)
LCST	Lower Critical Solution Temperature
PMEP	Poly(methyl ethylene phosphate)
Dopa	3,4-dihydroxyphenylalanine
PPEs	Polyphosphoesters
PEEP	Poly(ethyl ethylene phosphate)
AFM-CP	Atomic force microscopy–colloidal probe technique
ROP	Ring-opening polymerization
DBU	1,8-diazabicyclo(5.4.0)undec-7-ene
TU	1-(3,5-bis(trifluoromethyl)phenyl)-3-cyclohexylthiourea
PMEP MA	Poly(methyl ethylene phosphate) methacrylate
APTES	3-aminopropyl-triethoxysilane
BrIn	A-bromoisobutyryl bromide
ARGET-	Activators regenerated by electron transfer
ATRP	Atom transfer radical polymerization
PMDTA	*N*,*N*,*N*′,*N″*,*N″*-pentamethyldiethylenetriamine
NMR	Nuclear magnetic resonance
IEP	Isoelectric point
