# Supplementary material for: Thermo-Responsive Polymer Brushes with Side Graft Chains: Relationship Between Molecular Architecture and Underwater Adherence
Source: Int J Mol Sci. 2019 Dec 13;20(24):6295. doi: 10.3390/ijms20246295 (PMC6941113; doi:10.3390/ijms20246295)
Supplement: Supplementary file 1 [file ijms-20-06295-s001.pdf]

SUPPORTING INFORMATION

Thermo-responsive polymer brushes with side graft chains: Relationship between molecular architecture and underwater adherence

Ugo G. Sidoli[1,2], Hisaschi T. Tee[3], Ivan Raguzin[1], Jakob Mühldorfer[1,2], Frederick R. Wurm[3], Alla Synytska[1,2]*

[1] Leibniz-Institut für Polymerforschung Dresden e.V., Hohe Straße 6, 01069 Dresden, Germany

[2] Technische Universität Dresden, 01062 Dresden, Germany

[3] Max Planck Institute of Polymer Research, Ackermannweg 10, 55128 Mainz, Germany

*Corresponding author: Alla Synytska (AS) email: synytska@ipfdd.de

Contents:

NMR spectra of PNIPAm-PMEP 95:5 and 90:10
Calculations for grafting density (σ)
Influence of salt concentration on the cloud point of bulk polymer brushes
Representative curves for AFM-CP against SiO_2 CP – Debonding rate and contact time
Representative curves for AFM-CP against 1H,1H,2H,2H-Perfluorooctyldimethylchlorosilane CP – Debonding rate and contact time
Representative curves for AFM-CP against SiO_2 CP – Influence of salt concentration
Representative curves for AFM-CP against 3-Aminopropyltriethoxysilane CP – Influence of salt concentration
Representative curves for AFM-CP against 1H,1H,2H,2H-Perfluorooctyldimethylchlorosilane CP – Influence of salt concentration
Analysis of variation of contact angle) using PNIPAm-PMEP 95:5 as example

Number of pages: 14

Number of NMR spectra: 2

Number of figures: 9

NMR spectra for PNIPAm-PMEP copolymers

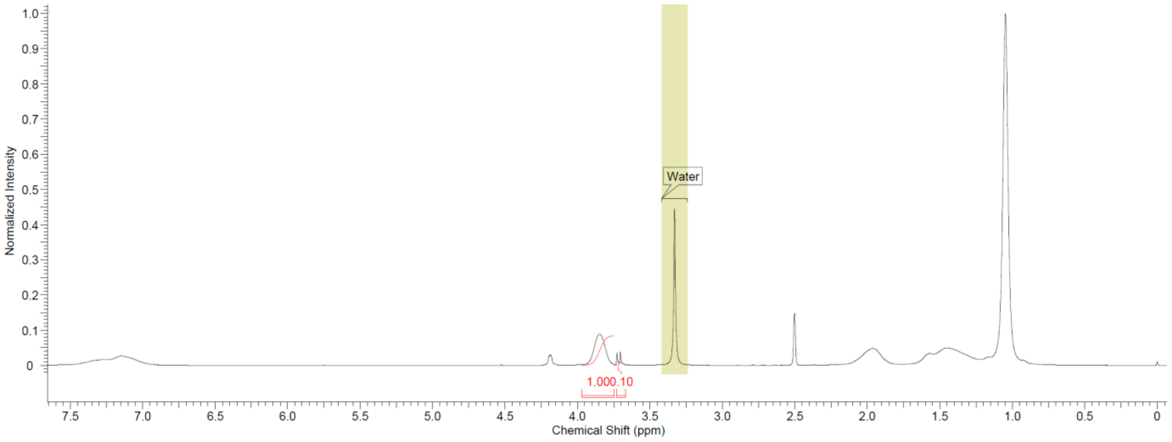

^{1}H NMR Spectrum for PNIPAm-PMEP 95:5

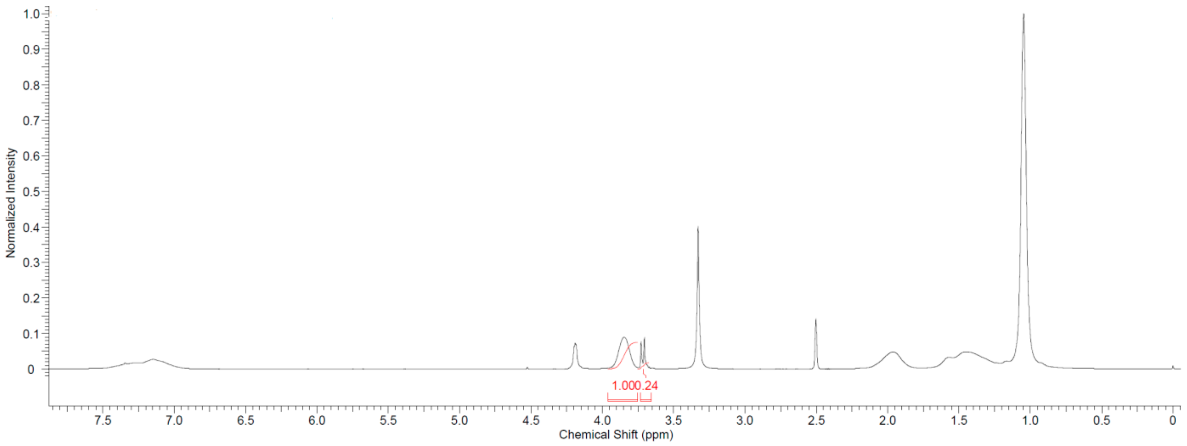

^{1}H NMR Spectrum for PNIPAm-PMEP 90:10

Calculations for grafting density (σ)

The grafting density (σ) is a key parameter for polymer brushes, because tethered polymer chains can adopt different conformations according to the attachment density. If the grafting density is too low, the chains will be far away from each other and the brushes will adopt the so-called "mushroom" conformation. If the density is increased to a certain critical value, the chains start to feel sterical repulsion and therefore they will stretch away from the surface to avoid this unfavorable interaction. This is the transition point between mushroom regime and brush regime[1]. The grafting density can be calculated by:

$$\sigma = \frac{h\,\rho\,Na}{Mn}$$

Where h is the dry thickness of the brushes, ρ is the mass density (here we assumed ρ = 1 g/cm3), Na is the Avogadro number and Mn is the molecular weight of the polymer. The grafting density is defined as the number of tethered chains per unit area. According to this definition, from the chain density also the distance between grafting points can be calculated by:

$$D = \sqrt{1/\sigma}$$

A first indication that our chains are in the brush regime can be obtained from the comparison between the distance between grafting points and the thickness of the layer, which is reported in **Error! Reference source not found.**:

Table S1. Values of grafting density (σ), distance between grafting points (D), molecular weight (Mn) and dry thickness of polymer brushes

**Ratio PNIPAm:Phosphate units**	**σ (chains/nm^2)**	**D (nm)**	**Mn (KDa)**	**dry thickness (nm)**
100:0	0.22	2.1	83	30.4 ± 0.2

95:5	0.26	1.8	55	35.1 ± 0.7
90:10	0.23	2.0	69	32.9 ± 0.9

Since the distance between grafted chains is one order of magnitude smaller compared to the thickness of the polymer film (2 nm compared to 30 nm), our layer can in first approximation be considered in the brush regime[2]. Another way to confirm the regime of brushes has been proposed by Minko and coworkers[3]. They proposed a single parameter, the reduced grafting density (Σ), to quantitatively describe the different regimes. The reduced grafting density can be calculated by:

$$\Sigma = \sigma \pi R_g^2$$

Where R_g is the radius of gyration of the polymer chain at specific conditions of solvent and temperature and σ is the grafting density. Physically, Σ indicates the number of chains occupying an area that a free polymer chain would fill. Therefore, this parameter is a good indicator of the brush-like behavior of thin films. In their paper, Minko and coworkers indicate three regimes for grafted films: 1) "mushroom" regime ($\Sigma < 1$); 2) crossover regime ($\Sigma = 1$); 3) highly stretched regime ($\Sigma > 1$). In reality, the polymer chains have a certain polydispersity and the grafting points can be distributed in a non-homogeneous way. For this reason, the transitions are not as sharp as theory predicts, and a "true brush" regime is characterized by $\Sigma > 5$. We measured the hydrodynamic radius (R_H) of the bulk polymers by DLS and we've calculated the radius of gyration (R_g) according to the relationship proposed by Akcasu and Han[4]:

$$R_H = 0.664\ R_g \quad \text{(theta solvent)}$$

$$R_H = 0.537\ R_g \quad \text{(good solvent)}$$

The calculated radius of gyration was 22.8 nm (theta solvent) and 28.2 nm (good solvent) for PNIPAm, 36.2 (theta solvent) and 43.7 nm (good solvent) for PNIPAm-g-PMEP 95:5, 38.1 nm

(theta solvent) and 46.5 nm (good solvent) for PNIPAm-g-PMEP. From these values we calculated the reduced grafting density, resulting in $\Sigma > 20$ for all systems either considering water a good or a theta solvent. We can therefore assume that our layers are in the "real brush" regime.

Influence of salt concentration on the cloud point of bulk polymer brushes

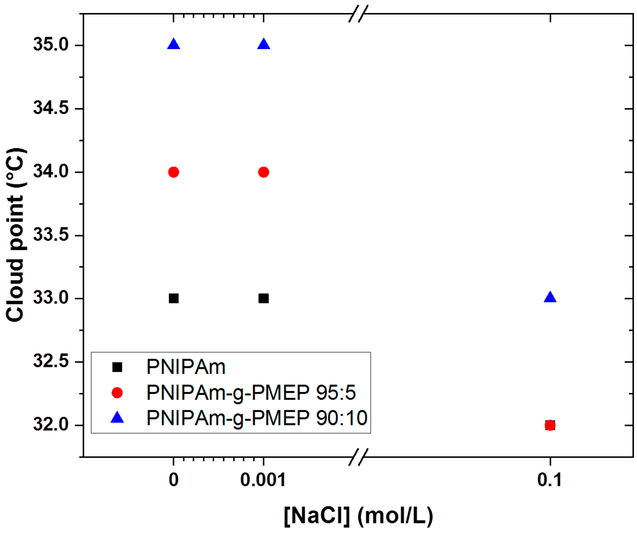

Figure S1. Cloud point of bulk polymer brushes measured via UV-vis spectroscopy (black: PNIPAm; red: PNIPAM-g-PMEP 95:5; blue: PNIPAm-g-PMEP 90:10)

Representative curves for AFM-CP against SiO2 CP – Debonding rate and contact time

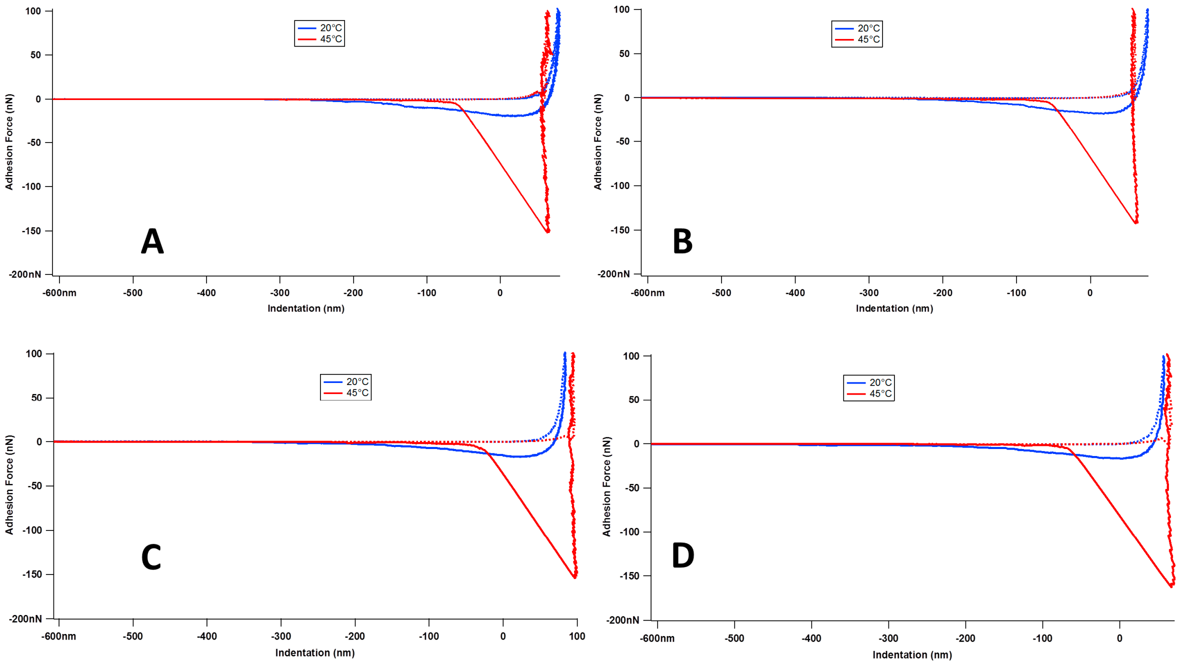

Figure S2. Force distance curves for debonding rate study against SiO_2 colloidal probe for PNIPAm-g-PMEP 90:10 at 10^{-3} M NaCl (blue: 20°C, red: 45°C); A= 300 nm/s; B= 600 nm/s; C= 1000 nm/s; D= 1500 nm/s

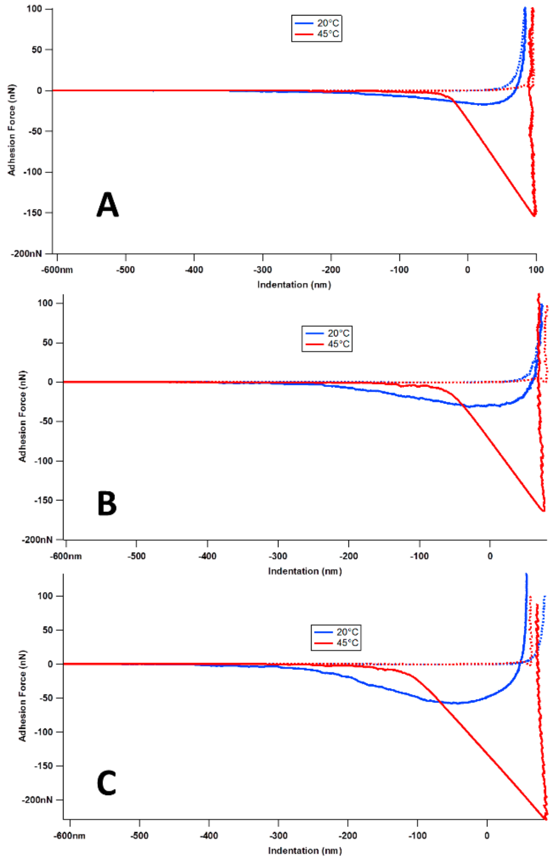

Figure S3. Force distance curves for contact time study against SiO_2 colloidal probe for PNIPAm-g-PMEP 90:10 at 10^{-3} M NaCl (blue: 20°C, red: 45°C); A= 0 s; B= 1 s; C= 10 s

Representative curves for AFM-CP against 1H,1H,2H,2H-Perfluorooctyldimethylchlorosilane CP – Debonding rate and contact time

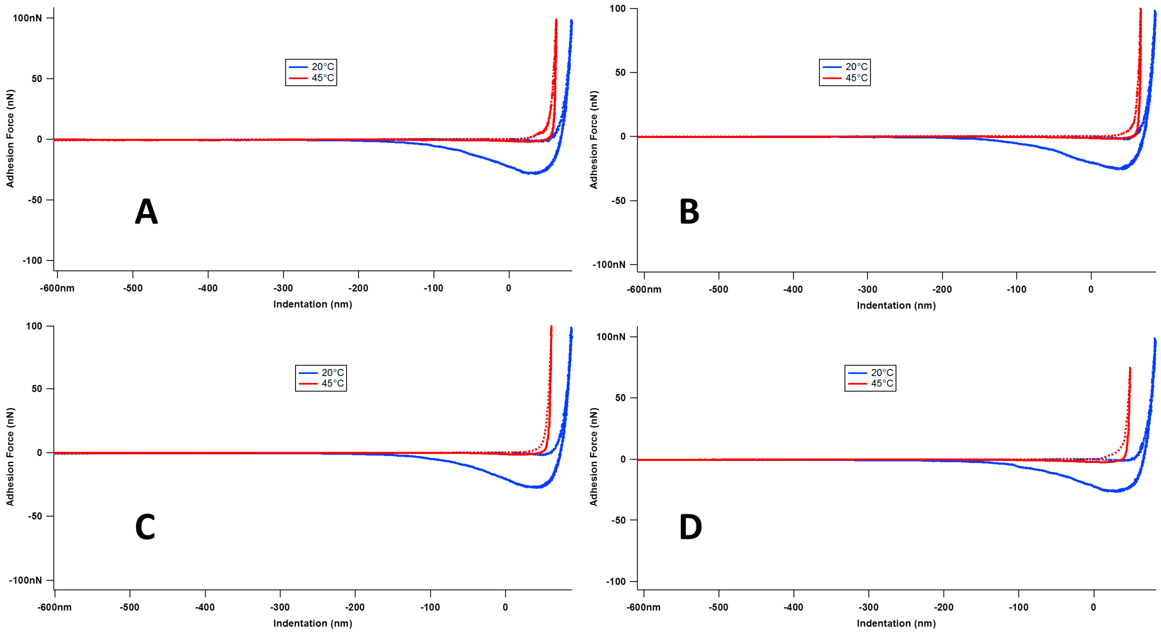

Figure S4. Force distance curves for debonding rate study against 1H,1H,2H,2H-Perfluorooctyldimethylchlorosilane colloidal probe for PNIPAm-g-PMEP 90:10 at 10^{-3} M NaCl (blue: 20°C, red: 45°C); A= 300 nm/s; B= 600 nm/s; C= 1000 nm/s; D= 1500 nm/s

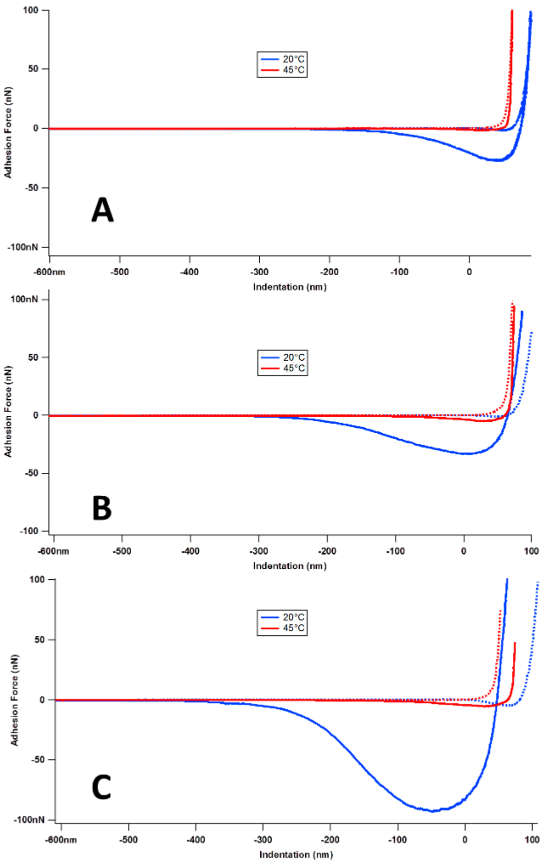

Figure S5. Force distance curves for contact time study against 1H,1H,2H,2H-Perfluorooctyldimethylchlorosilane colloidal probe for PNIPAm-g-PMEP 90:10 at 10^{-3} M NaCl (blue: 20°C, red: 45°C); A= 0 s; B= 1 s; C= 10 s

Representative curves for AFM-CP against SiO2 CP – Influence of salt concentration

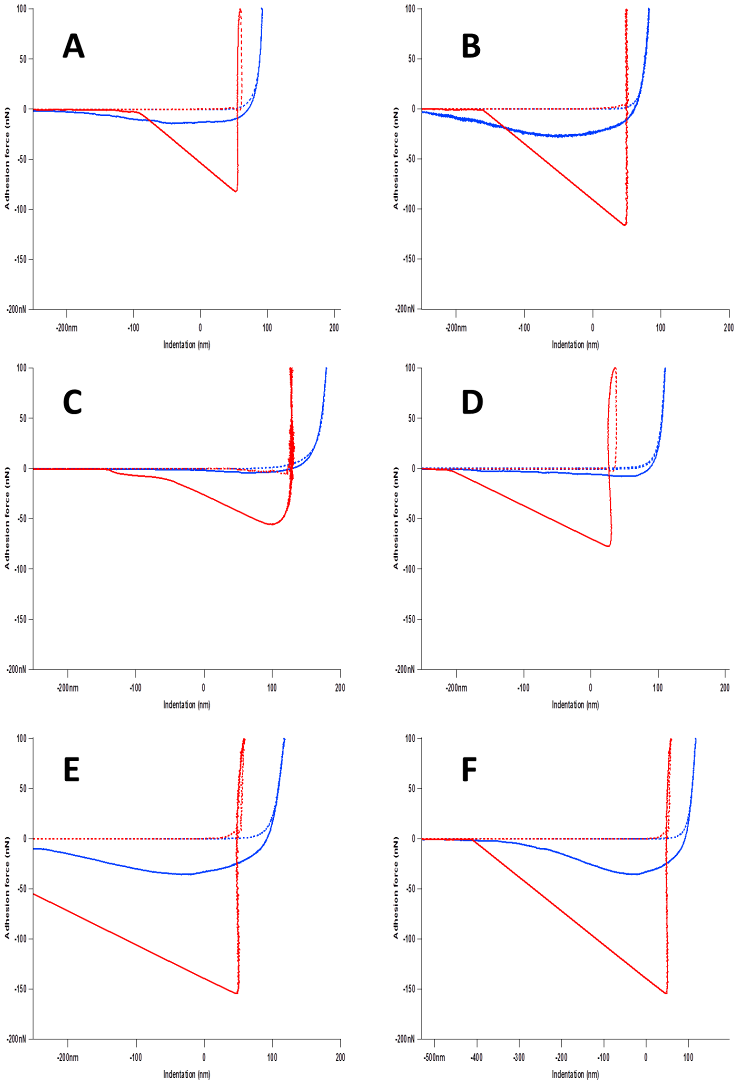

Figure S6. Force distance curves against SiO_2 colloidal probe for PNIPAm at 10^{-3} M NaCl (A) and 10^{-1} M NaCl (B), for PNIPAm-g-PMEP 95:5 at 10^{-3} M NaCl (C) and 10^{-1} M NaCl (D), for PNIPAm-g-PMEP 90:10 at 10^{-3} M NaCl (E) and 10^{-1} M NaCl (F) (blue line: measurement at 20°C; red line: measurement at 45°C)

Representative curves for AFM-CP against 3-Aminopropyltriethoxysilane CP – Influence of salt concentration

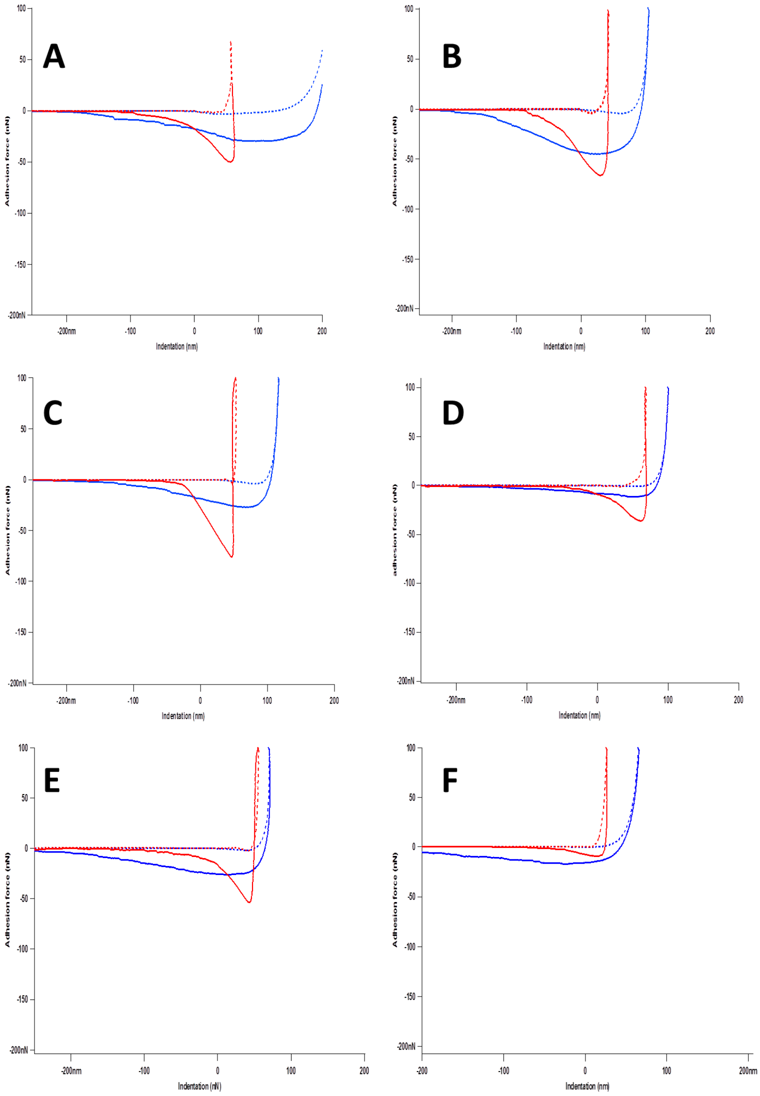

Figure S7. Force distance curves against amino-silane colloidal probe for PNIPAm at 10^{-3} M NaCl (A) and 10^{-1} M NaCl (B), for PNIPAm-g-PMEP 95:5 at 10^{-3} M NaCl (C) and 10^{-1} M NaCl (D), for PNIPAm-g-PMEP 90:10 at 10^{-3} M NaCl (E) and 10^{-1} M NaCl (F) (blue line: measurement at 20°C; red line: measurement at 45°C)

Representative curves for AFM-CP against 1H,1H,2H,2H-Perfluorooctyldimethylchlorosilane CP – Influence of salt concentration

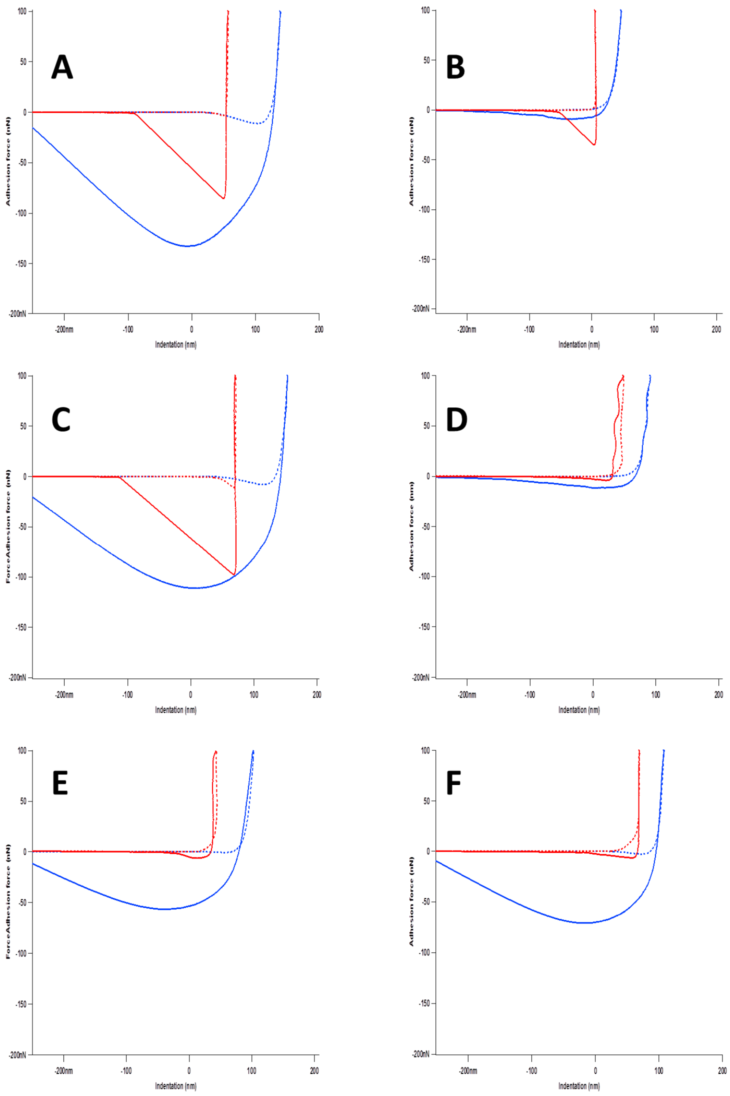

Figure S8. Force distance curves against fluorosilane colloidal probe for PNIPAm at 10^{-3} M NaCl (A) and 10^{-1} M NaCl (B), for PNIPAm-g-PMEP 95:5 at 10^{-3} M NaCl (C) and 10^{-1} M NaCl (D), for PNIPAm-g-PMEP 90:10 at 10^{-3} M NaCl (E) and 10^{-1} M NaCl (F) (blue line: measurement at 20°C; red line: measurement at 45°C)

Analysis of variation of contact angle using PNIPAm-PMEP 95:5 as example

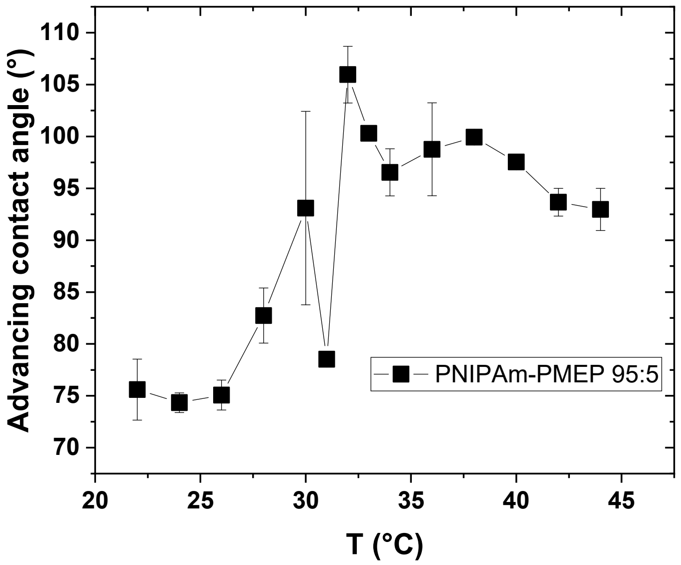

Figure S9. Variation of advancing contact angle for PNIPAm-PMEP 95:5 as function of temperature

PNIPAm films are known to change their wettability when the temperature reaches the LCST. Swollen hydrophilic chains undergo a coil-to-globule transition that pushes the contact angle to values corresponding to hydrophobic surfaces (angle > 90°C). In our systems, we can highlights two unexpected features. The copolymer PNIPAm-PMEP 95:5 is used as example to discuss these features. First of all, the advancing contact angle starts to increase with increasing temperature as expected but a sudden drop can be seen at 31°C. The contact angle goes from 93° at 30°C to 78° at 31°C, indicating that a first rearrangement takes place and also that this rearrangement seems to promote the presence of hydrophilic units at the interface. The value of contact angle then increases from 78° at 31°C to 105° at 32°C, indicating the complete collapse of the PNIPAm moieties which makes the surface hydrophobic as described in many studies in the literature. For T > 32°C a second rearrangement can be observed. The surface remains hydrophobic because the angle is > 90°, but the contact angle goes from 105° at 32° to 100° at 33° and then 96° at 34°C. This indicates that also during the second rearrangement more hydrophilic domains appear at the interface.

REFERENCES

1. Milner, S. T. Polymer brushes *Science* **1991,** 251, 905-914.

2. Synytska, A.; Svetushkina, E.; Puretskiy, N.; Stoychev, G.; Berger, S.; Ionov, L.; Bellmann, C.; Eichhorn, K.-J.; Stamm, M. Biocompatible polymeric materials with switchable adhesion properties. *Soft Matter* **2010,** 6 (23), 5907.
3. Brittain, W. J.; Minko, S. A structural definition of polymer brushes. *Journal of Polymer Science Part A: Polymer Chemistry* **2007,** 45 (16), 3505-3512.
4. Akcasu, A. Z.; Han, C. C. Molecular Weight and Temperature Dependence of Polymer Dimensions in Solution. *Macromolecules* **1979,** 12 (2), 276-281.
